# Complete mitochondrial genome of the Eastern slow worm, *Anguis colchica* (Nordmann, 1840)

**DOI:** 10.1080/23802359.2016.1275852

**Published:** 2017-02-06

**Authors:** Tomasz Strzała, Renata Grochowalska, Bartłomiej Najbar, Anna Najbar, Daniel Jablonski

**Affiliations:** a Department of Genetics, Wroclaw University of Environmental and Life Sciences, Wrocław, Poland;; b Department of Biochemistry and Bioinformatics, Faculty of Biological Sciences, University of Zielona Góra, Zielona Góra, Poland;; c Department of Botany and Ecology, Faculty of Biological Sciences, University of Zielona Góra, Zielona Góra, Poland;; d Department of Evolutionary Biology and Conservation of Vertebrates, University of Wrocław, Wrocław, Poland;; e Department of Zoology, Faculty of Natural Sciences, Comenius University in Bratislava, Bratislava, Slovakia

**Keywords:** *Anguidae*, legless lizards, mtDNA, mitogenome, comparative analysis

## Abstract

Here, we present complete mitochondrial genome of the Eastern Slow Worm, *Anguis colchica* (Nordmann, 1840). Mitogenome complete sequence is 17,097 bp long and consists of 13 protein-coding genes, 22 tRNA genes, two rRNA genes and one control region. *Anguis colchica* mitochondrial genome has the same gene order as other mitogenomes of *Anguis* spp. Their analyzed genome has base composition as: A (30.4%), T (24.6%), C (30.4%), G (14.6%), with an A + T bias (55%). Length of the all 22 tRNA genes varies from 65 to 73 bp with an average of 69 bp. Presented mitogenome will provide new data for phylogenetic analysis within the genus *Anguis.*

Genus *Anguis* is a small group of lizards from *Anguidae* family called slow worms. Recently, these legless lizards were under a very intensive study, which resulted with its taxonomic revisions (Gvoždík et al. 2010; Gvoždík et al. 2013). Based on the analyses of both mitochondrial and nuclear DNA fragments, Gvoždík et al. (2010) divided *A. fragilis* sensu lato into *A. fragilis* Linnaeus, 1758*, A. colchica* (Nordmann, 1840) and *A. graeca* Bedriaga, 1881 (Gvoždík et al. 2010). Furthermore, the same genetic tools proved the presence of fifth slow worm species, *A. veronensis* Pollini, 1818, endemic for Apennine Peninsula (Gvoždík et al. 2013). Further analyses confirmed earlier studies and showed distinctness of the new species and their deep intraspecific variability (Mezzasalma et al. 2013; Jablonski et al. 2016).

Although *A. colchica* originated by split from *A*. *fragilis* sensu lato (former subspecies; see Gvoždík et al. 2010) it belongs to relatively slightly known species. Although genetic data clearly separated *Anguis* spp. into five taxa, morphological differences did not confirm these findings (Gvoždík et al. 2010; Gvoždík et al. 2013). According to Gvoždík et al. (2013) and Jablonski et al. (2016) the species occurs mainly in eastern parts of Europe, northern Anatolia, western Russia, Transcaucasian region and northern Iran. Thus, to provide more comprehensive data for phylogenetic analyses, we sequenced complete mitochondrial DNA (MtDNA) genome of *A. colchica* representative.

Total genomic DNA was isolated from a road-killed individual (adult male collected in Zatwarnica village, the Bieszczady Mts.; SE Poland; 49°13’47.5" N 22°33’20.1" E) with Sherlock AX (A&A Biotechnology, Gdynia, Poland) according to producers instructions. Analysed individual is deposited in the Faculty of Biological Sciences collection under voucher number AC 148, at the University of Zielona Góra. MtDNA complete genome was amplified with three overlapping fragments using PCR and then sequenced. Sequencing was carried out by Wyzer Biosciences (Cambridge, MA) using primer walking method. Genome assembling was done using MITOS WebServer (Bernt et al. 2013) and checked manually. The sample taxonomical status was confirmed with phylogenetic tree created with Bayesian inference using MrBayes 3.2.5 (Figure 1) (Ronquist et al. 2012).

**Figure 1.**
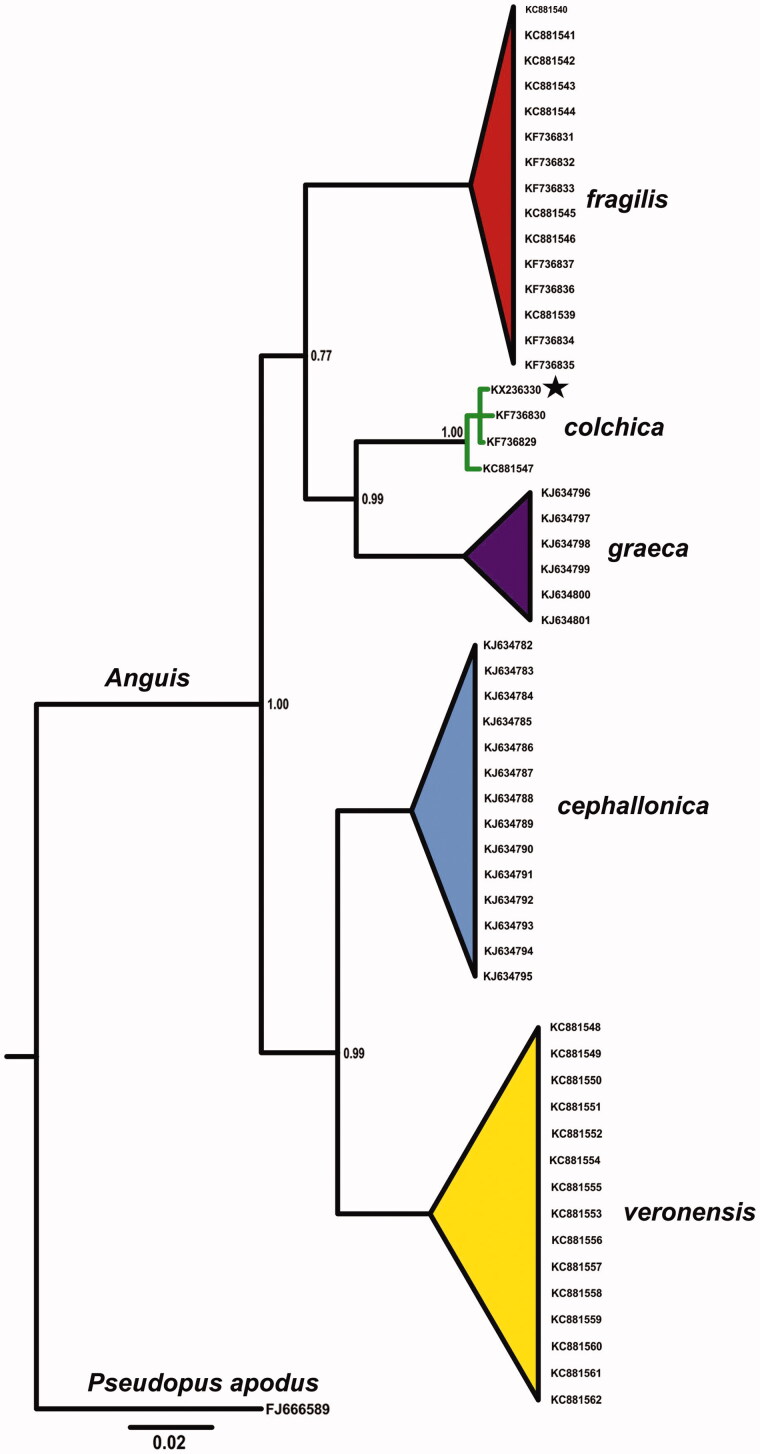
Bayesian phylogenetic tree of *Anguis* spp. representatives, created with 765 bp of ND2 gene alignment. The tree was created using GTR + I +G model of substitution, as suggested by jModelTest 2.1.10 (Guindon & Gascuel 2003; Darriba et al. 2012). Tree was generated with 10,000,000 MCMC generations and 25% of burn-in. The individual used for complete mitochondrial genome is marked by star. A homologous sequence of *Pseudopus apodus* (Pallas, 1775) was used as outgroup. GenBank accession numbers and Bayesian posterior probabilities of nodes are shown on the tree.

The sequence of *A. colchica* complete mitochondrial genome had 17,097 bp (KX236330) and revealed the same gene order as other representatives of *Anguis* spp. Analysed genome was shorter than mtDNA of *A. cephallonica* (17,208 bp) and *A. fragilis* (17,479 bp). When compared to *A. fragilis* mitogenome, *A. colchica* had the same start and stop codons for all the protein-coding genes (PCGs), while comparison with *A. cephallonica* showed differences in one start codon (ND3 gene: GTG in *A. colchica* vs. ATG in *A. cephallonica*) and five stop codons (ND1 (TAA), ND2 (TAG), COX1 (TAG), ATP6 (TAX), ND5 (TXX) for *A. colchica* vs. ND1 (TAG), ND2 (TAA), COX1 (TAA), ATP6 (TAA), ND5 (TAA) for *A. cephallonica*). In *A. colchica* mitogenome four PCGs have used truncated stop codons (COX2, COX3, ND3, ND4). The analysed species H-strand overall base composition is as follows: A (30.4%), T (24.6%), C (30.4%), G (14.6%), with an A + T bias (55%).
